# A method for continuous noninvasive assessment of respiratory mechanics during spontaneous breathing

**DOI:** 10.1186/cc10724

**Published:** 2012-03-20

**Authors:** K Lopez-Navas, S Brandt, H Gehring, M Strutz, U Wenkebach

**Affiliations:** 1 Fachhochschule Lübeck, Germany; 2 Universitätsklinikum Schleswig-Holstein, Lübeck, Germany

## Introduction

The proper assessment of patient's work of breathing (WOB) is the key to a better or even automatic setting of ventilation parameters. We introduce the Occlusion+Delta method (O+D) to continuously determine resistance (R) and compliance (C), allowing one to assess noninvasively the inspiratory force.

## Methods

The O+D method uses a short expiratory occlusion producing immediate changes in airway pressure (Paw), flow (V') and volume (V) but not in transdiaphragmatic pressure (Pdi). The differences between an occluded and an undisturbed cycle are related by V'R + V/C = Paw + Pdi. If both cycles are similar Pdi can be neglected, making its measurement unnecessary. Then R and C are derived from linear regression (MLR) and used to make a reconstruction of Pdi (rPdi). As control, R and C were calculated by MLR using the objectively measured (with balloon catheters) Pdi. The inspiratory pressure time product (PTPinsp) of measured Pdi (APdi) and reconstructed Pdi (ArPdi) were compared as expression of WOB.

## Results

**Figure 1:**
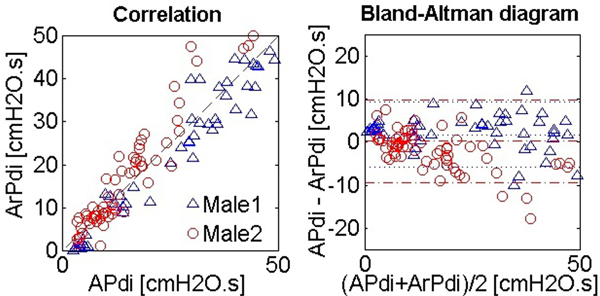
**PTPinsp from measured Pdi (APdi) versus PTPinsp from reconstruction (ArPdi)**.

After validation with simulations, we used data from two healthy adults breathing at several levels of WOB. The occlusions caused the expected signals reproducing Pdi as desired with R and C values typical for healthy men (Table 1). Measured and assessed PTPinsp correlated well (*R*^2 ^= 0.93 and 0.89) and had small mean differences (mean ± 2SD = 1.78 ± 3.81 and 0.27 ± 4.80 cmH_2_O.second) (Figure 1).

**Table 1:** 

	Male 1	Male 2
R estimated	3.7 ± 0.7	3.2 ± 0.7
R measured	5.2 ± 1.9	2.9 ± 1.2
C estimated	97.7 ± 20.6	85.4 ± 18.7
C measured	100.5 ± 21.9	76.5 ± 18.7

Mean ± SD of R in cmH_2_O/l/second and C in ml/mbar (measured = MLR, estimated = O+D).

## Conclusion

Our first results demonstrate a great potential in the proposed method. A study with 30 volunteers is being carried out and results will be presented in 2012.

